# Activation of Rac1 Has an Opposing Effect on Induction and Maintenance of Long-Term Potentiation in Hippocampus by Acting on Different Kinases

**DOI:** 10.3389/fnmol.2021.720371

**Published:** 2021-08-31

**Authors:** Dongyang Cui, Xiaodong Jiang, Ming Chen, Huan Sheng, Da Shao, Li Yang, Xinli Guo, Yingqi Wang, Bin Lai, Ping Zheng

**Affiliations:** ^1^State Key Laboratory of Medical Neurobiology, Institutes of Brain Science, MOE Frontier Center for Brain Science, School of Basic Medical Sciences, Fudan University, Shanghai, China; ^2^Department of Neurology of Zhongshan Hospital, Fudan University, Shanghai, China; ^3^Department of Pharmacology of Medical College of China Three Gorges University, Yichang, China

**Keywords:** Rac1, long term potentiation, PI3K, LIMK, PKCι/λ, PKMζ

## Abstract

Rac1 is a small GTPase of the Rho family. A previous study showed that the activation of Rac1 had an opposing effect on induction and maintenance of long-term potentiation (LTP) in the hippocampus. However, the molecular mechanism underlying this opposing effect remains to be addressed. In the present work, we find that the activation of Rac1 during the induction of LTP leads to an activation of PKCι/λ by phosphatidylinositol-3-kinase (PI3K), whereas the activation of Rac1 during the maintenance of LTP leads to the inhibition of PKMζ by LIM_kinase (LIMK) in the hippocampus. This result suggests that during different stages of LTP, the activation of Rac1 can modulate different signaling pathways, which leads to an opposing effect on the induction and maintenance of LTP in the hippocampus.

## Introduction

Small GTPases are important signaling molecules in neurons. One of the best characterized subfamilies of the small GTPases is the Rho family, which includes Rac, Cdc42, and Rho (Hall, 2005). Among them, Rac1 has been reported to be involved in morphological plasticity in the hippocampus. Rac1 could induce spine morphogenesis and synapse formation in the hippocampus (Luo, 2000; Tolias et al., 2005). In addition, Rac1 also participates in functional plasticity in the hippocampus. Martinez and Tejada-Simon (2011) reported that the induction of long-term potentiation (LTP) in the hippocampus was coupled with the activation of Rac1 and the inhibition of Rac1 suppressed the induction of LTP in a dose-dependent manner (Martinez and Tejada-Simon, 2011). Interestingly, during the maintenance phase of LTP in the hippocampus, it appeared that the activation of Rac1 had an opposing effect on LTP. Liu et al. reported that the application of an adeno-associated virus that carried transgene to activate Rac1 during the maintenance phase of LTP resulted in an accelerated LTP decay in the hippocampus (Liu et al., 2016). However, the molecular mechanism underlying the opposing effect of the activation of Rac1 on the induction and maintenance of LTP in the hippocampus remains to be addressed.

Typical downstream signal transduction pathway of the activation of Rac1 includes two steps: first, the activation of p21-activated kinase (Pak); second, the Pak-induced activation of LIM-domain-containing protein kinase (LIM-kinase), which subsequently phosphorylates and inhibits cofilin, an actin depolymerization factor, thus inducing actin polymerization (Luo, 2000). However, it is hard to explain the opposing effect of the activation of Rac1 on the induction and maintenance of LTP using this typical downstream signal transduction pathway of Rac1.

It has been known that numerous signaling molecules have been involved in the induction and maintenance of LTP (Baltaci et al., 2019). Among them, Wang et al. (2016) reported that phosphorylated protein kinase C iota/lambda (pPKCι/λ) showed a marked increase during the induction phase of LTP but returned to the control level during the maintenance phase of LTP, whereas PKMζ increased significantly only during the maintenance phase of LTP (Wang et al., 2016). Using a recombinant adeno-associated virus (rAAV2/8) expressing small hairpin RNA (shRNA) that targeted the gene of either PKCι/λ or PKMζ, it was found that the knockdown of PKCι/λ produced a reduction in the early expression of LTP during the induction phase, whereas the knockdown of PKMζ disrupted only the late phase of LTP during the maintenance phase (Wang et al., 2016). These pieces of evidence suggest that the activation of PKCι/λ played an important role in the induction of LTP, while the activation of PKMζ played a key role in the maintenance of LTP. Therefore, we proposed a hypothesis that Rac1 might have an opposing effect on PKCι/λ and PKMζ, thus producing an opposing effect on the induction and maintenance of LTP in the hippocampus. To test this hypothesis, using electrophysiological method combined with the Western blotting and pharmacological approaches, we studied the role of the activation of Rac1 during the induction and maintenance phases of LTP in the hippocampus and further explored the downstream signaling pathways of Rac1 activation during the induction and maintenance phases of LTP.

## Results

### Activation of Rac1 Has an Opposing Effect on the Induction and Maintenance of LTP in Hippocampal CA1

**Figure 1:**
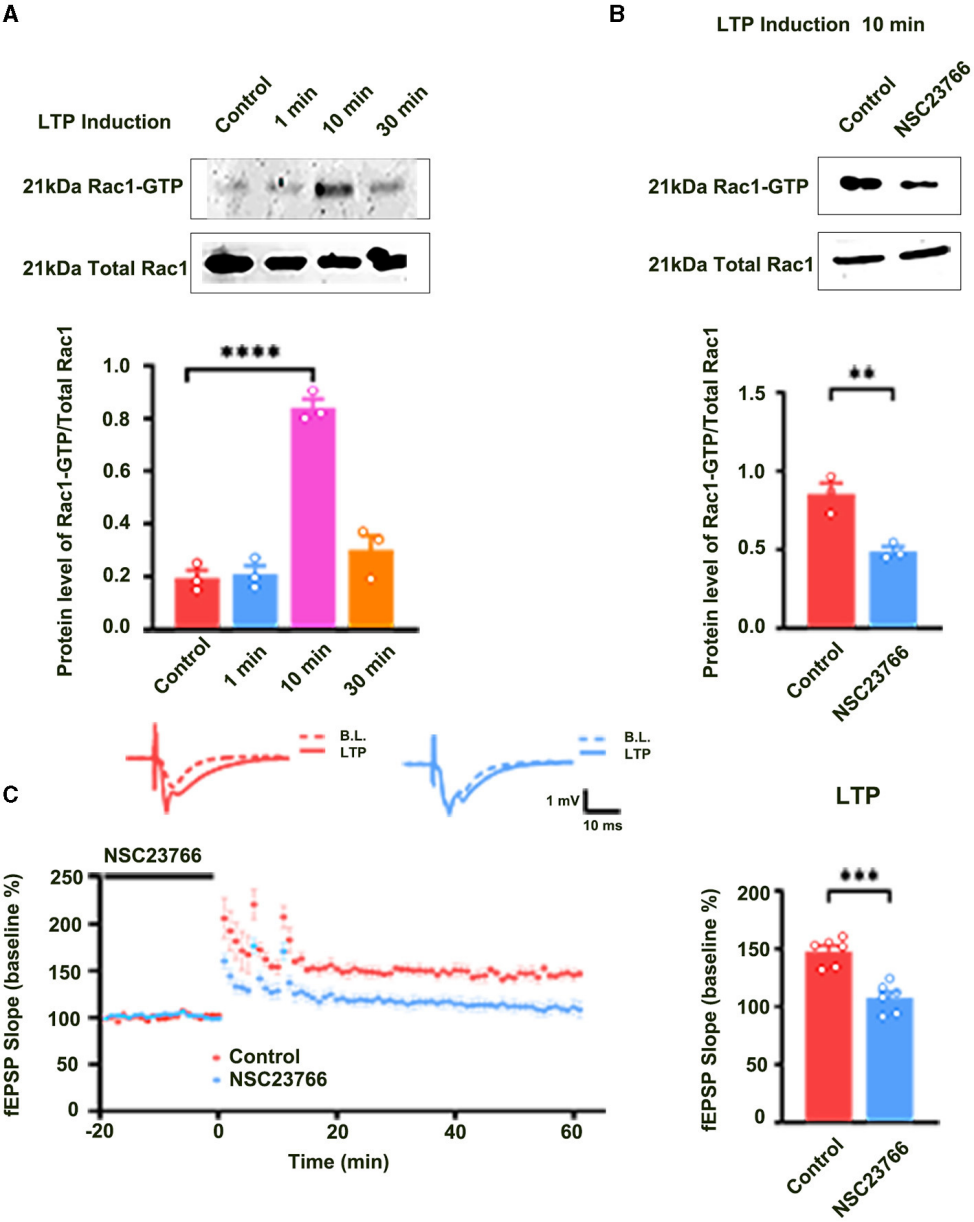
The role of Rac1 in LTP induction. **(A)** Influence of three trains of HFS on Rac1 activation (one-way ANOVA, ****P < 0.0001, *n* = 3 in each group). **(B)** Influence of Rac1 antagonist NSC23766 (100 μM) on Rac1 activation at 10 min after HFS (unpaired *t-*test, **P < 0.01, *n* = 3 in each group). **(C)** Influence of Rac1 antagonist NSC23766 (100 μM) on LTP induction (unpaired *t-*test, ***P < 0.001, *n* = 6 in each group). Top: Typical fEPSP trace at baseline and during LTP recordings. Left: LTP was recorded for 1 h after three trains of HFS. Right: Average fEPSP slope during the last 15 min of the LTP recording. All data are shown as mean ± SEM.

Long-term potentiation (LTP) is generally divided into at least two distinct phases: the induction phase and the maintenance phase (Baltaci et al., 2019). To evaluate the role of Rac1 in the induction of LTP, we first detected whether Rac1 was activated during the induction phase of LTP. Rats were divided into four groups: one group was the control group where Hippocampal CA1 slices were not given high-frequency stimulation (HFS),and the other three groups were divided into 1-min group, 10-min group, and 30-min group based on the time duration after giving HFS. The result showed that the level of the activation state of Rac1 (Rac1-GTP) was significantly increased at 10 min after HFS (one-way ANOVA*, F*_(3, 8)_ = 63.24, control group, 0.19 ± 0.029, *n* = 3; 10-min group, 0.84 ± 0.033, *n* = 3; *P* < 0.0001; Figure 1A), but returned to control level at 30 min after HFS (one-way ANOVA, 30-min group, 0.30 ± 0.055, *n* = 3, vs. control group; *P* = 0.1974; Figure 1A). This result suggests that LTP induction is associated with a transient activation of Rac1. We then studied the role of Rac1 activation in LTP induction by examining the influence of Rac1-specific inhibitor NSC23766 (Martinez and Tejada-Simon, 2011) on the LTP induction. Rats were divided into two groups: control group where artificial cerebrospinal fluid (ACSF) was applied at 30 min before HFS, and NSC23766 group where NSC23766 (100 μM) was applied at 30 min before HFS. The result showed that after the application of NSC23766, Rac1 activation by HFS at 10 min after HFS was significantly inhibited when compared with the control group (unpaired *t-*test, *t*_(4)_ = 4.893, control group, 0.85 ± 0.068, *n* = 3; NSC23766 group, 0.49 ± 0.029, *n* = 3; *P* = 0.0081; Figure 1B), and LTP induction by HFS was also significantly inhibited by NSC23766 (unpaired *t-*test, *t*_(10)_ = 5.592, control group, 146.7 ± 4.1%, *n* = 6; NSC23766 group, 108.3 ± 8.8%, *n* = 6; *P* = 0.0027; Figure 1C). These results suggest that Rac1 activation contributes significantly to LTP induction.

**Figure 2:**
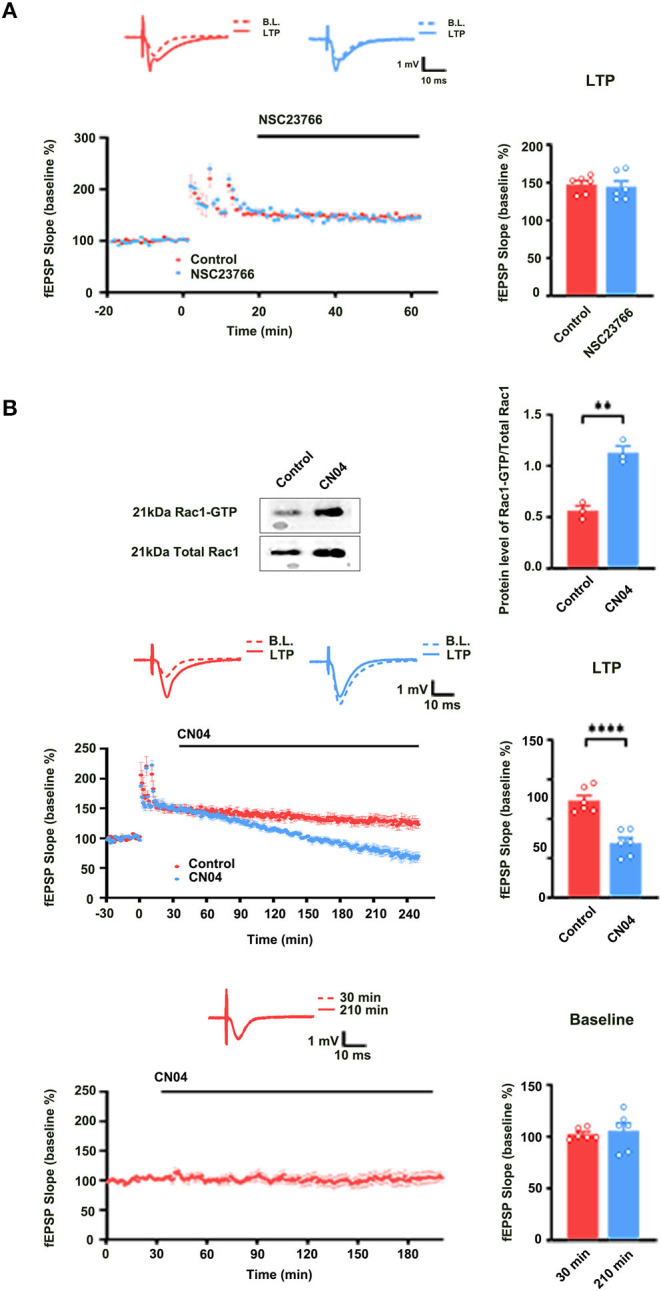
The role of Rac1 in LTP maintenance. **(A)** Influence of Rac1 antagonist NSC23766 (100 μM) on the LTP maintenance (Unpaired *t-*test, *P* > 0.05, *n* = 6 in each group). **(B)** Top: Influence of Rac1 agonist CN04 (424 nM) on Rac1 activation (unpaired *t-*test, **P < 0.01, *n* = 3 in each group). Top right: Average fEPSP slope during the last 15 min of the LTP recording. Middle: Influence of Rac1 agonist CN04 (424 nM) on the LTP maintenance (unpaired *t-*test, ****P < 0.0001, *n* = 6 in each group). Middle right: Average fEPSP slope during the last 15 min of the LTP recording. Bottom: Influence of Rac1 agonist CN04 (424 nM) on the fEPSP baseline (unpaired *t-*test, *P* > 0.05, *n* = 6 in each group). All data are shown as mean ± SEM.

We also evaluated the contribution of Rac1 activation to LTP maintenance by examining the influence of Rac1-specific inhibitor NSC23766 on the LTP maintenance. NSC23766 was applied 10 min after the last HFS. Result showed that LTP maintenance was unaffected after the application of NSC23766 (unpaired *t-*test, *t*_(10)_ = 0.3290, control group, 139.0 ± 5.9%, *n* = 6; NSC23766 group, 138.1 ± 7.7%, *n* = 6, *P* = 0.7489; Figure 2A). This result suggests that Rac1 activation does not contribute to LTP maintenance. Interestingly, when we applied Rac1 agonist CN04 (Jiang et al., 2016) during the maintenance phase of LTP, CN04 induced an accelerated decay of LTP. The rats were divided into two groups: control group where ACSF was applied and CN04 group where CN04 (424 nM) was applied. The top panel of Figure 2B shows that in a normal brain slice containing the CA1 region, 90 min treatment with CN04 could significantly increase the level of Rac1-GTP (unpaired *t-*test, *t*_(4)_ =7.044, control group, 0.56 ± 0.047, *n* = 3; CN04 group, 1.13 ± 0.065, *n* = 3; *P* = 0.0021; Top panel of Figure 2B). The middle and bottom panels of Figure 2B show the effect of CN04 on LTP maintenance and baseline of field excitatory post-synaptic potentials (fEPSPs). We could see that CN04 treatment resulted in an accelerated LTP decay during the maintenance phase (unpaired *t-*test, *t*_(10)_ = 5.831, control group, 123.2 ± 6.4%, *n* = 6; CN04 group, 69.1 ± 6.7%, *n* = 6; *P* = 0.0002; The middle panel of Figure 2B), whereas the treatment had no influence on baseline of fEPSPs (unpaired *t-*test, *t*_(10)_ = 5.592, 30 min control group, 97.3 ± 4.5%, *n* = 6; 210 min CN04 group, 107.0 ± 5.8%, *n* = 6; *P* = 0.2239; The bottom panel of Figure 2B). CN04 is not a specific activator of Rac1 agonist, as it also activates CDC42 and RhoA. So the influence of CN04 on LTP may be due to Rac1, CDC42, or RhoA. To confirm the CN04-induced decrease in LTP maintenance was mediated by Rac1 activation, the Pak1, a specific downstream molecular of Rac1 pathway, was inhibited by IPA-3 before CN04 treatment. The rats were divided into two groups: CN04 group where CN04 (424 nM) was applied, and IPA-3 + CN04 group where the Pak1 inhibitor IPA-3 (100 μM) and CN04 were co-applied. Supplementary Figure 1 shows that Pak1 inhibitor IPA-3 could reverse CN04-induced decrease in LTP maintenance (unpaired *t-*test, *t*_(10)_ = 3.270, CN04 group, 69.1 ± 6.7%, *n* = 6; IPA-3 + CN04 group, 116.4 ± 7.7%, *n* = 6; *P* = 0.0084; The bottom panel of Figure 2B), suggesting that CN04-induced decrease in LTP maintenance was related to Rac1 activation. These results suggest that the activation of Rac1 during the maintenance phase accelerates LTP decay.

### Activation of Rac1 During the Induction Phase of LTP Results in Activation of PKCι/λ Through PI3K Pathway in the Hippocampus

**Figure 3:**
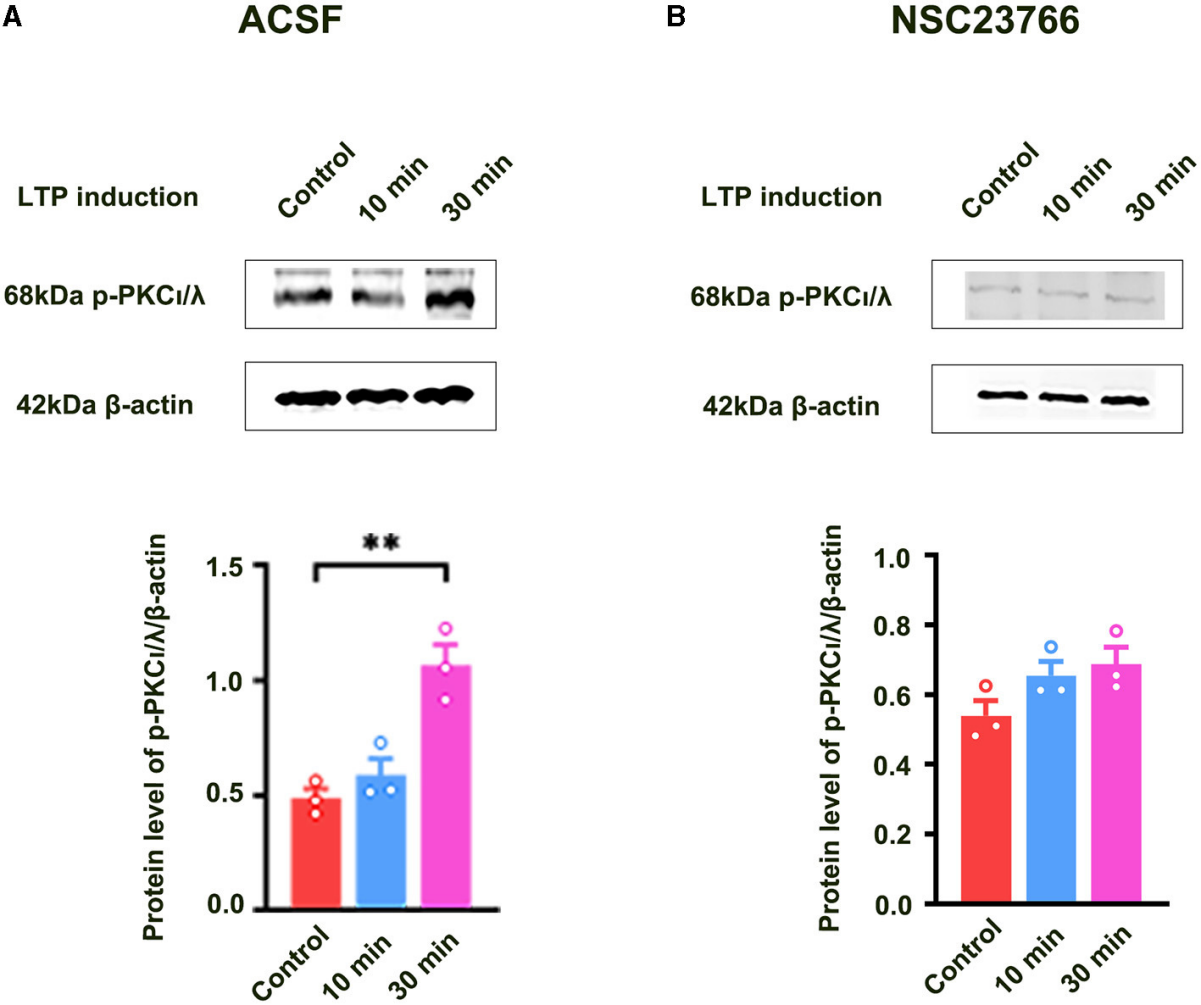
Influence of Rac1 inhibitor NSC23766 on HFS-induced activation of PKCι/λ. **(A)** Influence of three trains of HFS on PKCι/λ activation (one-way ANOVA, **P < 0.01, *n* = 3 in each group). **(B)** Influence of Rac1 antagonist NSC23766 (100 μM) on PKCι/λ activation after giving HFS (one-way ANOVA, *P* > 0.05, *n* = 3 in each group). All data are shown as mean ± SEM.

Previous study showed that PKCι/λ was activated during the induction phase of LTP and the knockdown of PKCι/λ could inhibit LTP induction (Wang et al., 2016). However, the manner in which PKCι/λ is activated during the induction phase of LTP remains to be addressed. To evaluate whether Rac1 activation was an upstream mechanism of the activation of PKCι/λ during the induction phase of LTP, we examined the influence of Rac1 inhibitor NSC23766 on HFS-induced activation of PKCι/λ. The rats were divided into three groups:one group was the control group where the slices were not given HFS,and the other two groups were divided into 10- min group and 30-min group according to the time duration after giving HFS. Figure 3A shows HFS-induced activation of PKCι/λ (one-way ANOVA, *F*_(2, 6)_ = 19.48, control group, 0.49 ± 0.041, *n* = 3; 30-min group, 1.06 ± 0.090, *n* = 3; *P* = 0.0020; Figure 3A). Figure 3B shows the influence of NSC23766 on HFS-induced activation of PKCι/λ. We could see that after the application of NSC23766, HFS-induced activation of PKCι/λ was inhibited (one-way ANOVA, *F*_(2, 6)_ = 3.02, control group, 0.54 ± 0.043, *n* = 3; 30-min group, 0.69 ± 0.049, *n* = 3; *P* = 0.1233; Figure 3B). This result suggests that Rac1 activation is an upstream mechanism of the activation of PKCι/λ during the induction phase of LTP.

**Figure 4:**
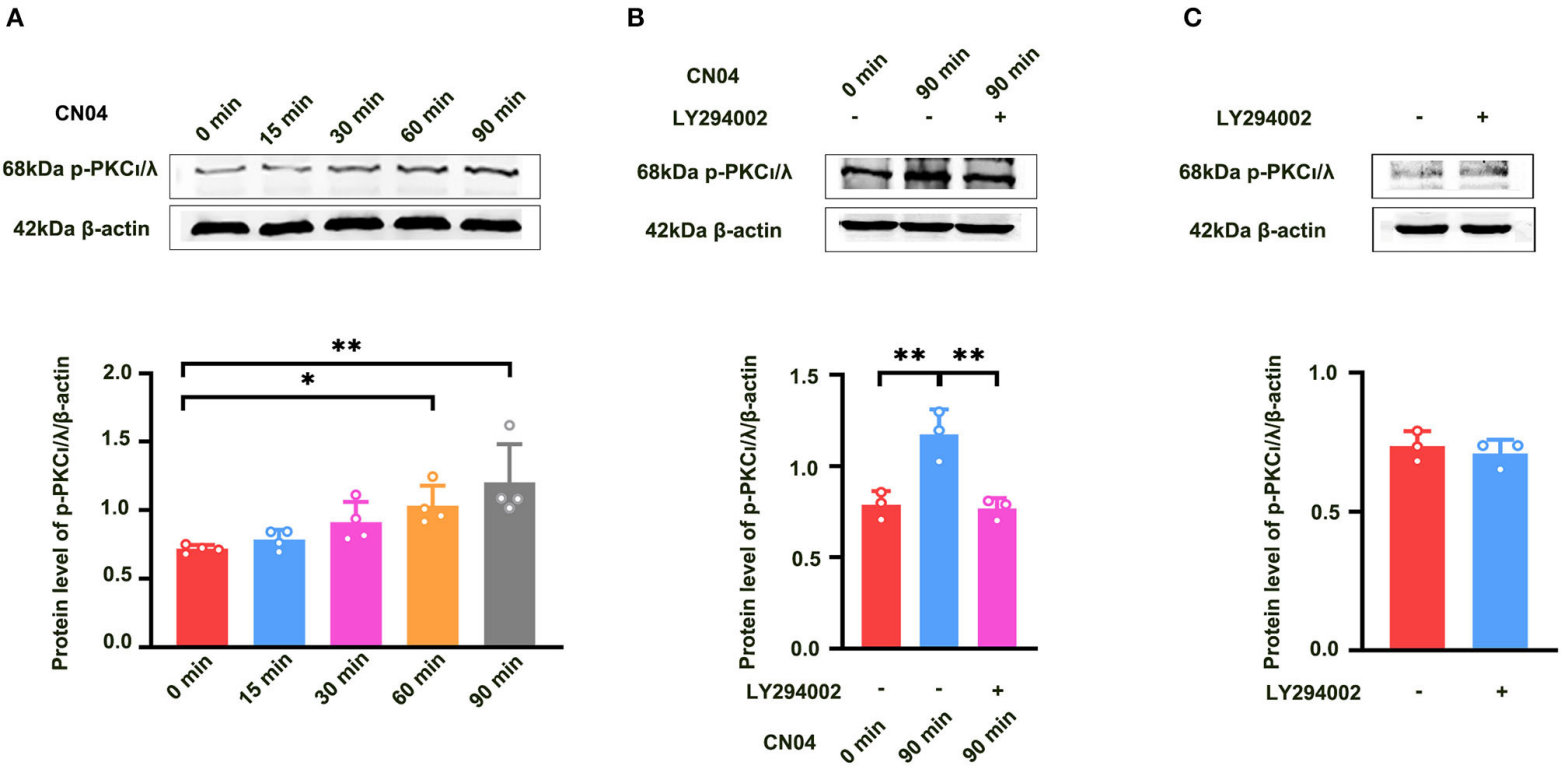
Influence of P13K inhibitor LY294002 on Rac1-induced activation of PKCι/λ. **(A)** Influence of Rac1 agonist CN04 (424 nM) on PKCι/λ activation (one-way ANOVA, *P < 0.05, **P < 0.01, *n* = 4 in each group). **(B)** Influence of P13K inhibitor LY294002 (100 μM) on the effects of Rac1 agonist CN04 (424 nM) on PKCι/λ activation (one-way ANOVA, **P < 0.01, *n* = 3 in each group). **(C)** Influence of P13K inhibitor LY294002 (100 μM) on PKCι/λ activation (unpaired *t-*test, *P* > 0.05, *n* = 3 in each group). All data are shown as mean ± SEM.

We further studied how Rac1 activation results in the activation of PKCι/λ. We examined the influence of the specific P13K inhibitor LY294002 (Hsueh et al., 2015) on Rac1-induced activation of PKCι/λ. Firstly, we examined Rac1 agonist CN04-induced activation of PKCι/λ. The rats were divided into five groups:one group was control group where ACSF was applied, and the other four groups were divided into 15-min group, 30-min group, 60-min group, and 90-min group according to the duration of the application of CN04 (424 nM). Figure 4A shows Rac1 agonist CN04-induced activation of PKCι/λ (one-way ANOVA, *F*_(4, 15)_ = 5.899, control group, 0.72 ± 0.014, *n* = 4; 60-min group, 1.03 ± 0.073, *n* = 4, vs. control group; *P* = 0.0443; 90-min group, 1.20 ± 0.140, *n* = 4, vs. control group; *P* = 0.0023; Figure 4A). This result suggests that CN04 indeed can induce the activation of PKCι/λ. We then examined the influence of LY294002 on Rac1-induced activation of PKCι/λ. The rats were divided into three groups:control group where ACSF was applied, CN04 group where CN04 (424 nM) was applied, and CN04 + LY294002 group where CN04 (424 nM) and LY294002 (100 μM) were co-applied. Figure 4B shows the influence of LY294002 on Rac1-induced activation of PKCι/λ. We could see that after the application of LY294002, Rac1-induced activation of PKCι/λ was inhibited (one-way ANOVA, *F*_(2, 6)_ = 16.96, CN04 group, 1.17 ± 0.079, *n* = 3; LY294002 + CN04 group, 0.77 ± 0.033, *n* = 3; *P* = 0.0037; Figure 4B), whereas LY294002 treatment had no influence on basis of PKCι/λ activation (unpaired *t-*test, *t*_(4)_ = 0.6160, control group, 0.74 ± 0.031, *n* = 3; LY294002 group, 0.71 ± 0.028, *n* = 3; *P* = 0.5712; Figure 4C). These results suggest that Rac1 activation may result in the activation of PKCι/λ through PI3K pathway in the hippocampus.

### Activation of Rac1 During the Maintenance of LTP Results in the Inhibition of PKMζ Through LIMK Pathway in the Hippocampus

**Figure 5:**
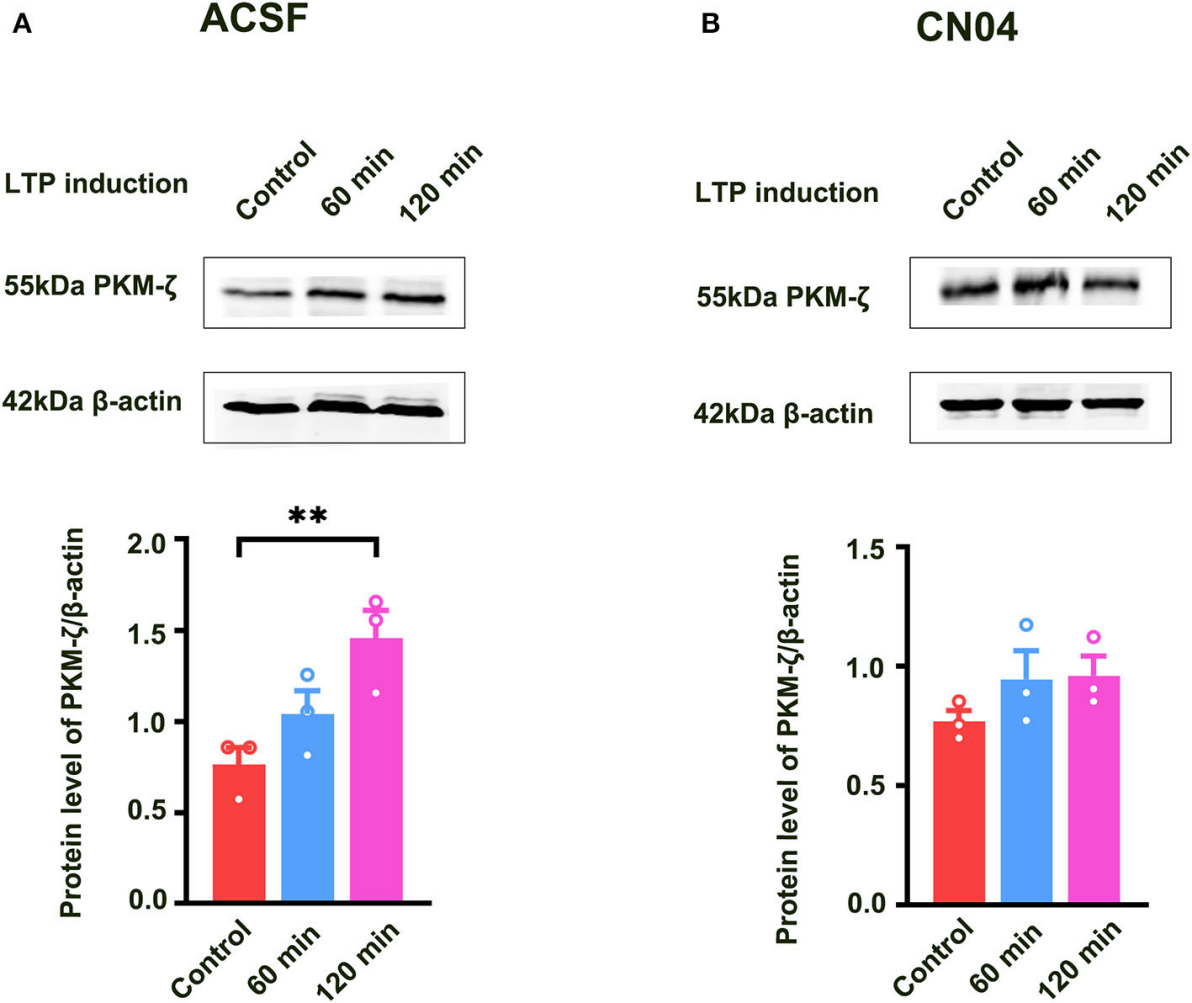
Influence of Rac1 agonist CN04 on HFS-induced activation of PKMζ. **(A)** Influence of three trains of HFS on PKMζ expression (one-way ANOVA, **P < 0.01, *n* = 3 in each group). **(B)** Influence of Rac1 agonist CN04 (424 nM) on PKMζ expression after giving HFS (one-way ANOVA, *P* > 0.05, *n* = 3 in each group). All data are shown as mean ± SEM.

Previous study showed that PKMζ was a key molecule for the maintenance of LTP (Baltaci et al., 2019). It is unclear whether Rac1-induced LTP decay during the maintenance phase is related to the inhibition of the expression of PKMζ. To address this question, we examined the effect of a specific Rac1 agonist CN04 on increased expression of PKMζ during the maintenance phase of LTP. The rats were divided into three groups:one group was control group where slices were not given HFS,and the other two groups were divided into 60-min group and 120-min group based on the time duration after giving HFS. Figure 5A shows that the expression of PKMζ showed a significant increase at 120 min after LTP induction (one-way ANOVA, *F*_(2, 6)_ = 14.83, control group, 0.80 ± 0.061, *n* = 3; 120-min group, 1.52 ± 0.088, *n* = 3; *P* = 0.0031; Figure 5A). Figure 5B shows the influence of CN04 on the increased expression of PKMζ during the maintenance phase of LTP. We could see that after the application of CN04, the expression of PKMζ did not change at 60 and 120 min after LTP induction, compared with the control group (one-way ANOVA, *F*_(2, 6)_ = 1.480, control group, 0.77 ± 0.045, *n* = 3; 120-min group, 0.96 ± 0.082, *n* = 3; *P* = 0.3002; Figure 5B). This result suggests that PKMζ is a downstream molecule of Rac1 activation during the maintenance phase of LTP.

**Figure 6:**
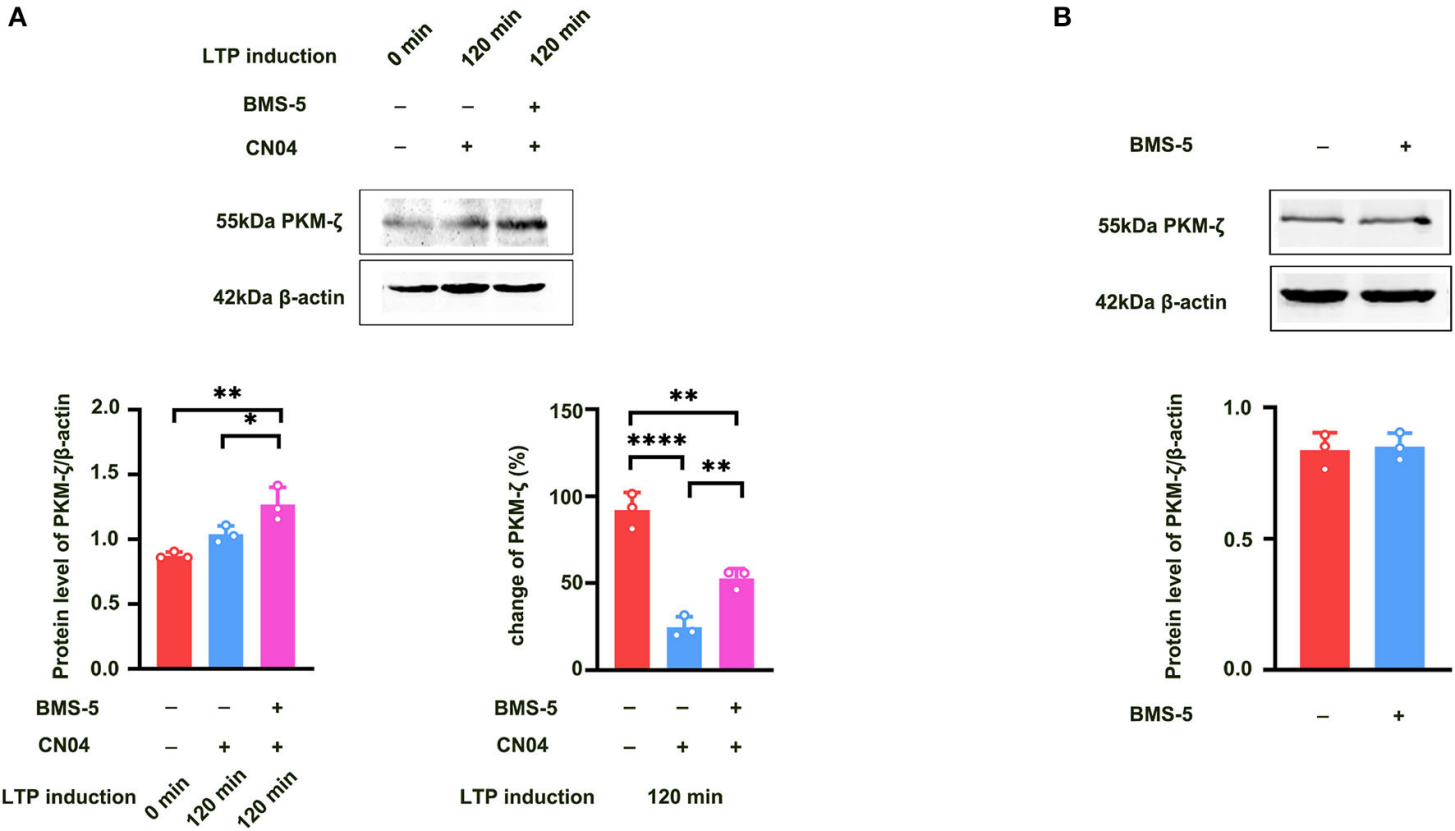
Influence of LIMK inhibitor BMS-5 on Rac1-induced decrease in the expression of PKMζ. **(A)** Influence of LIMK inhibitor BMS-5 (100 μM) on the effects of Rac1 agonist CN04 (424 nM) on PKMζ expression at 120 min after giving HFS (Bottom left: one-way ANOVA, *P < 0.05, **P < 0.01, *n* = 3 in each group). Bottom right: The bar graph shows the change of PKMζ expression from 0 min to 120 min after LTP induction (one-way ANOVA, **P < 0.01, ****P < 0.0001, *n* = 3 in each group; The control group data is calculated using the data of Figure 5A). **(B)** Influence of LIMK inhibitor BMS-5 (100 μM) on PKMζ expression (unpaired *t-*test, *P* > 0.05, *n* = 3 in each group). All data are shown as mean ± SEM.

We further studied how Rac1 activation results in a decrease in the expression of PKMζ. We examined the influence of a specific LIMK inhibitor BMS-5 (Lunardi et al., 2018) on Rac1-induced decrease in the expression of PKMζ. The rats were divided into three groups:a control group where ACSF was applied, CN04 group where CN04 was applied, and CN04 + BMS-5 group where CN04 and BMS-5 were co-applied. Result showed that after the application of BMS-5, Rac1-induced decrease in the expression of tetanization-induced PKMζ was reversed (one-way ANOVA, *F*_(2, 6)_ = 60.83, CN04 group, 24.6 ± 3.5%, *n* = 3; control group, 92.1 ± 5.8%, *n* = 3, vs. CN04 group *P* < 0.0001; BMS-5+CN04 group, 52.8 ± 3.2%, *n* = 3, vs. CN04 group; *P* = 0.0067; Figure 6 bottom right), whereas the BMS-5 treatment had no influence on the basis of the expression of PKMζ (unpaired *t-*test, *t*_(4)_ = 0.2662, control group, 0.84 ± 0.038, *n* = 3; BMS-5 group, 0.85 ± 0.030, *n* = 3; *P* = 0.8033; Figure 6B). This result suggests that Rac1-induced LTP decay during the maintenance phase is related to the inhibition of the expression of PKMζ in the hippocampus.

## Discussion

Previous studies have examined the role of the activation of Rac1 in LTP. Martinez and Tejada-Simon (2011) reported that the induction of LTP in the hippocampus was coupled with the activation of Rac1 in area CA1 and the inhibition of Rac1 suppressed the induction of LTP in a dose-dependent manner (Martinez and Tejada-Simon, 2011). This result is consistent with our current conclusion that Rac1 participates in the induction of LTP in the hippocampus. This statement was also supported by the findings that upon induction of structural LTP (sLTP) using uncaging of glutamate on single spines, an activation of Rac1 was observed and the addition of Rac1 inhibitor before sLTP induction effectively inhibited sLTP. However, the statement in this paper that the persistent activation of Rac1 was required for the maintenance of sLTP still lacked the evidence because the activation of Rac1 was observed only for 33 min and not for a longer duration. Moreover, Martinez and Tejada-Simon (2011) reported that the activity of Rac1 showed a transient increase during the induction of LTP, but returned to the control level during the maintenance phase of LTP (Saneyoshi et al., 2019). This result is consistent with our current statement that the level of Rac1 activity is rather low during the maintenance phase of LTP. This statement is also supported by the result that Rac1 activity is significantly elevated in the hippocampal tissues of adult mice in response to 7-day social isolation, but decreases to a low level after resocialization (Liu et al., 2018). In contrast, Liu et al. showed that there might be an activation of Rac1 during the maintenance phase of LTP because after the application of adeno-associated viruses (AAVs) that carried transgene to inhibit endogenous Rac1 activity, they found that LTP decay during the maintenance phase significantly decreased (Liu et al., 2016). The reason for the difference in Rac1 activation during the maintenance phase of LTP in different studies remains unknown. However, most of the results from literature (Liu et al., 2016, 2018) and our current study show that the activation of Rac1 during the maintenance phase of LTP in the hippocampus resulted in an accelerated LTP decay. The activator of Rac1 that we used here was CN04 that could also activate CDC42 and RhoA, and hence the influence of CN04 on LTP maintenance may be due to Rac1, CDC42, or RhoA. In order to determine whether CN04-induced decrease in LTP maintenance was related to Rac1 activation, we included an experiment on the influence of the inhibition of specific downstream target Pak1 (Lv et al., 2013) on CN04-induced decrease in LTP maintenance. The result showed that the Pak1 inhibitor IPA-3 could reverse CN04-induced decrease in LTP maintenance, suggesting that CN04-induced decrease in LTP maintenance was related to Rac1 activation.

It is interesting to study the reason behind why the activation of Rac1 during the induction and maintenance phases of LTP has an opposing effect in the hippocampus. In a typical downstream signal transduction pathway of the activation of Rac1, Rac1 first activates Pak, which then activates LIM-kinase, resulting in the phosphorylation of cofilin, thus inducing an actin polymerization. This pathway partly explains the mechanism underlying the participation of Rac1 in the induction of LTP because Rac1-induced actin polymerization results in an enlargement of dendritic spines, which leads to enhanced trapping of AMPA receptors in the postsynaptic membrane and potentiated synaptic transmission (Baltaci et al., 2019). In addition, Tolias et al. (2005) reported that Rac1 could associate with phosphoinositide 3-kinase (PI3K), whereas Ren et al. (2013) reported that LTP induced by PI3K activation could be significantly attenuated by PKCι/λ inhibitor Myr-aPKC-PS and the activation of PI3K could activate PKCι/λ (Ren et al., 2013). Therefore, it is possible that the activation of Rac1 during the induction phase of LTP leads to an activation of PKCι/λ by PI3K pathway in the hippocampus. This hypothesis was confirmed by our present result that the inhibition of PI3K could attenuate Rac1-induced activation of PKCι/λ. Since it has been known that PKCι/λ activation is required for both GluA1 phosphorylation and increased surface expression of AMPA receptors during the induction of LTP, it is possible that in addition to Rac1-induced Pak-LIMK-actin polymerization-enlarged dendritic spines pathway, PI3K-PKCι/λ-GluA1 phosphorylation / increased AMPA receptors may be another downstream signal pathway of the activation of Rac1 to participate in the induction of LTP. In addition, based on the already known upstream signaling pathway of Rac1 (Saneyoshi et al., 2019), the likely core signaling of early-LTP is: NMDAR to Ca2+ to CaMKII to Rac1 to PI3K to PKCι/λ to AMPA receptors. However, it is hard to explain the opposing effect of the activation of Rac1 on the maintenance of LTP using these two Rac1-induced pathways.

Molecules and signaling pathways mediating the maintenance of LTP have been identified in previous sections (Baltaci et al., 2019). Among them, the continuous enzymatic effect of the constitutively active PKMζ is thought to be the key molecule in the maintenance of LTP (Sacktor, 2011). Wang et al. (2016) reported that after 30 min of LTP induction, PKCι/λ significantly increased but then returned to control level 2 h after LTP induction, whereas PKMζ significantly increased after 2 h of LTP induction (Wang et al., 2016). In the meantime, LTP induction-induced increase in the Rac1 activity also returned to control level during the maintenance phase of LTP (Martinez and Tejada-Simon, 2011). Therefore, it appears that Rac1 does not participate in the maintenance of LTP. However, if exogenous activation of Rac1 during the maintenance phase of LTP, the activated Rac1 could inhibit LTP (Liu et al., 2016, 2018 and present result). Obviously, this inhibition is not due to the re-activation of PKCι/λ by Rac1 because the role of the activation of PKCι/λ is to potentiate synaptic transmission. Thus, we proposed that it was possible that the activation of Rac1 during the maintenance phase of LTP inhibited LTP via the suppression of PKMζ. This statement was supported by our present result that the activation of Rac1 during the maintenance phase of LTP could suppress PKMζ.

Positive feedback-like mechanism has been proposed to provide PKMζ required to maintain LTP (Baltaci et al., 2019). The PKMζ mRNA is carried to the dendrites following transcription, but its translation is inhibited by a peptidyl–prolyl isomerase PIN1. Following LTP induction, the activity of PIN1 diminishes and its suppressive effect on PKMζ translation ceases, and then PKMζ performs synthesis during the maintenance phase of LTP. So there are two possible ways by which the activation of Rac1 inhibits PKMζ: one is the inhibition of PKMζ transcription and the other is the inhibition of PKMζ translation. Obviously, the typical downstream signal transduction pathway of Rac1 through LIM-kinase-cofilin-actin cannot explain the inhibitory effect of Rac1 on PKMζ because this pathway is not related to the transcription and translation of proteins. Thus, alternative downstream pathways independent of cofilin-actin of Rac1 should be considered. Yang et al. (2004) reported that LIM-kinase could directly phosphorylate cAMP-responsive element-binding protein (CREB), which led to the stimulation of subsequent gene transcription (Yang et al., 2004) and participated in the maintenance of LTP (Todorovski et al., 2015). Ramos et al. reported that when phospho-CREB was increased in the aged prefrontal cortex, further stimulation of this pathway, even with a very low dose of an activator could exacerbate memory deficits (Ramos et al., 2003). These pieces of evidence suggest that if phospho-CREB already increases, further stimulation of this pathway may accelerate the decay of the maintenance of LTP, which exacerbates memory deficits. This statement was supported by the result that there was an activation of LIM-kinase accompanied by an increase in CREB during the maintenance phase of LTP (Todorovski et al., 2015) and that the further activation of LIM-kinase by Rac1 during the maintenance phase of LTP accelerated the decay of the maintenance of LTP. However, it is still unclear how over-activated CREB inhibits the transcription or translation of PKMζ.

In conclusion, the present results showed that the activation of Rac1 during the induction of LTP leads to an activation of PKCι/λ by PI3K, whereas the activation of Rac1 during the maintenance of LTP leads to the inhibition of PKMζ by LIMK in the hippocampus. These results suggest that during different stages of LTP, the activation of Rac1 can modulate different signaling pathways, which leads to an opposing effect on the induction and maintenance of LTP in the hippocampus.

## Star Methods

### Key Resources Table (KRT)

**Table:** 

**REAGENT or RESOURCE**	**SOURCE**	**IDENTIFIER**
Antibodies		
anti-Rac1	Pierce	Cat#16118
anti-β-actin	Santa Cruz	Cat#sc-47778
anti-p-PKCι/λ (Thr555/563)	Abcam	Cat#ab-5813
anti-PKCζ (c-20)	Santa Cruz	Cat#sc-216
Medicine		
NSC23766	Tocris	Cat#2161
Rho/Rac/cdc42 Activator I (CN04)	Cytoskeleton	Cat#CN04-B
LY294002	MedchemExpress	Cat#9901s
IPA-3	Tocris	Cat#3622
BMS-5	MedchemExpress	Cat#HY-18305
Experimental Models: Organisms/Strains		
Wildtype Sprague Dawley rats, male	JSJ Biotech	N/A
Software and Algorithms		
Prism 10.7	GraphPad	https://www.graphpad.com
Adobe Photoshop CS6	Adobe	https://www.adobe.com
Clampfit 10.7	Axon	N/A
Other		
active Rac1 Pull-Down and Detection Kit	Pierce	Cat#16118

## Experimental Model and Subject Details

Male adult (6–8 weeks) Sprague-Dawley rats were housed singly in a 12 h light/dark cycle in a temperature- and humidity-controlled environment with food and water freely available. All experimental procedures conformed to Fudan University as well as the international guidelines on the ethical use of animals. All efforts were made to minimize animal suffering and reduce the number of animals used.

## Method Details

### Slice Preparation and Electrophysiology

Hippocampal slices (400 μm) were prepared from 8-week-old rats using a vibratome (Leica) (Leutgeb et al., 2003). Slices were incubated in 32°C oxygenated artificial cerebrospinal fluid (ACSF) containing 124 mM NaCl, 3 mM KCl, 1.25 mM KH_2_PO_4_, 1 mM MgSO_4_, 2 mM CaCl_2_, 26 mM NaHCO_3_, and 10 mM glucose (pH 7.2–7.4) for at least 2 h before recording. Slices were placed in a recording chamber and perfused by oxygen-saturated ACSF with a flow rate of 4–5 ml/min. Extracellular field excitatory post-synaptic potentials (fEPSPs) in the Schaffer Collateral pathway were synaptically evoked at 0.017 Hz and recorded in the CA1 region. The fEPSPs were evoked using a stimulation intensity that elicited a 40% maximal response. LTP was induced by three trains of high-frequency stimulation (HFS, 1 s at 100 Hz spaced 5 min apart). The stimulation during the HFS was the same strength as test stimulation. The fEPSPs were recorded with an Axopatch700B amplifier (Axon) connected to a Digidata1440 interface (Axon). Data acquisition and analysis were performed using the Axon software packages Clampfit.

### Western Blot Analysis

Four brain slices containing the hippocampal CA1 region were homogenized in a buffer containing 100 mM Tris-HCl (pH=6.7), 1% SDS, 143 mM 2-mercaptoethanol, and 1% protease inhibitor. The lysate was centrifuged at 12,000 rpm for 10 min at 4°C. The samples were treated with the SDS sample buffer at 100°C for 10 min, loaded on a 10% SDS polyacrylamide gel, and blotted to a nitrocellulose (NC) membrane. The membranes were blocked for 1 h at room temperature in a blocking solution (Beyotime, China), followed by incubation overnight at 4°C with various primary antibodies that included anti-Rac1 at a dilution of 1:500; anti-pPKCι/λ, anti-PKMζ, and anti-β-actin at a dilution of 1:1,000. Afterward, the membranes were rinsed with 1 × TBST (Sangon, China) for three times (5 min for each wash), followed by incubation respectively with IRDye 680 LT goat anti-rabbit secondary antibody (1:10,000) and IRDye 800 CW goat anti-mouse secondary antibody (1:10,000) for 1 h at room temperature. Finally, after rinsing the membranes for three times (5 min for each wash) with 1 × TBST, we acquired the images with LI-COR Odyssey system.

### Assay for GTPase Activity

Active Rac1 pull-down was performed as described by the commercial active Rac1 Pull-Down and the Detection Kit protocol (Pierce, catalog #16118). Briefly, lysates of the rat hippocampal CA1 tissue were centrifuged at 16,000 *g* at 4°C for 15 min, and then the supernatants were transferred to a new tube, and GTPYS or GDP was added and incubated at 30°C for 15 min under the condition of constant agitation. The mixtures were then incubated with glutathione resin beads and glutathione S-transferase-fused Rac-binding domain of p21-activated kinase (Pak) at 4°C for 1 h; the beads had been washed several times previously to remove nonspecific binding. The beads and proteins bound to the fusion protein were washed three times with wash buffer at 4°C, eluted in SDS sample buffer, and analyzed for bound Rac1 by Western blotting using anti-Rac1.

## Quantification and Statistical Analysis

Statistical significance was determined using unpaired *t*-test for comparisons between two groups or ANOVAs for comparisons among three or more groups. All of the statistical details of the experiments can be found in the results. In all cases, *n* refers to the number of animals. Graphpad Prism 8.4 was used to process and analyze data and make statistical graphs. Data are presented as mean ± SEM.

## Data Availability Statement

The original contributions presented in the study are included in the article/Supplementary Material, further inquiries can be directed to the corresponding authors.

## Ethics Statement

The animal study was reviewed and approved by The Animal Care and Use Committee of Shanghai Medical College of Fudan University.

## Author Contributions

DC was responsible for conception and design, acquisition of data, analysis, interpretation of data, and drafting or revising the article. XJ, MC, HS, DS, LY, XG, and YW were responsible for the acquisition of data and analysis and interpretation of data. BL was responsible for conception and design and analysis and interpretation of data. PZ was responsible for conception and design, analysis and interpretation of data, and drafting or revising the article. All authors contributed to the article and approved the submitted version.

## Conflict of Interest

The authors declare that the research was conducted in the absence of any commercial or financial relationships that could be construed as a potential conflict of interest.

## Publisher's Note

All claims expressed in this article are solely those of the authors and do not necessarily represent those of their affiliated organizations, or those of the publisher, the editors and the reviewers. Any product that may be evaluated in this article, or claim that may be made by its manufacturer, is not guaranteed or endorsed by the publisher.

## Supplementary Material

Supplementary Figure 1 Influence of Pak1 inhibitor IPA-3 on CN04-induced decrease of LTP maintenance.Influence of adding Pak1 inhibitor IPA-3 (100 μM) before the application of CN04 (424 nM) on the CN04-induced decrease of LTP maintenance (unpaired *t-*test, ^**^*P* < 0.01, *n* = 6 in each group). All data are shown as mean ± SEM.

Supplementary Figure 2 The full-length immunoblot.
