# Supplementary figures and images for: Activation of Rac1 Has an Opposing Effect on Induction and Maintenance of Long-Term Potentiation in Hippocampus by Acting on Different Kinases
Source: Front Mol Neurosci. 2021 Aug 31;14:720371. doi: 10.3389/fnmol.2021.720371 (PMC8438208; doi:10.3389/fnmol.2021.720371)

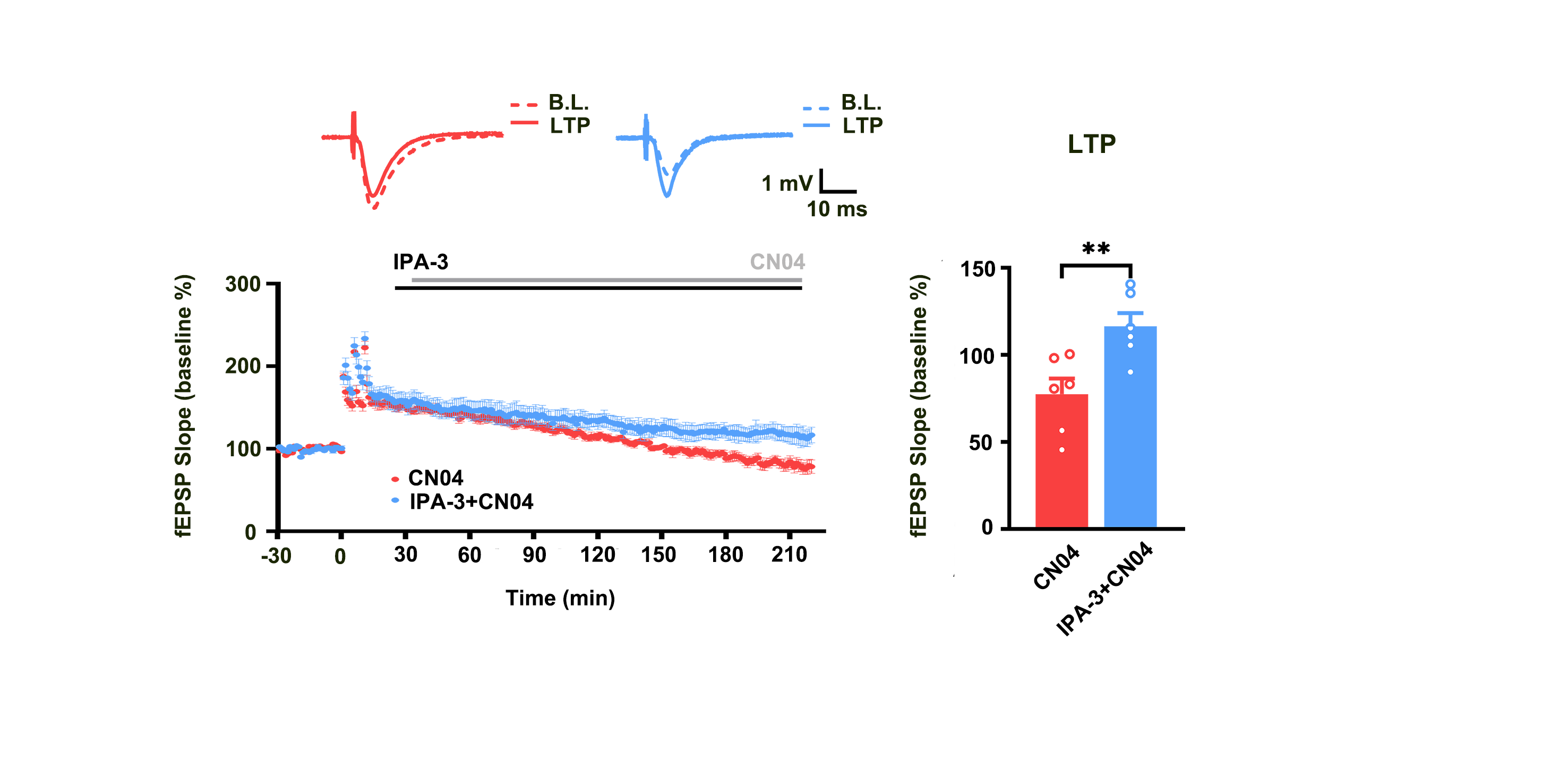

Supplement: Supplementary Figure 1 — Influence of Pak1 inhibitor IPA-3 on CN04-induced decrease of LTP maintenance.Influence of adding Pak1 inhibitor IPA-3 (100 μM) before the application of CN04 (424 nM) on the CN04-induced decrease of LTP maintenance (unpaired t-test, **P < 0.01, n = 6 in each group). All data are shown as mean ± SEM. [file Image_1.TIF]

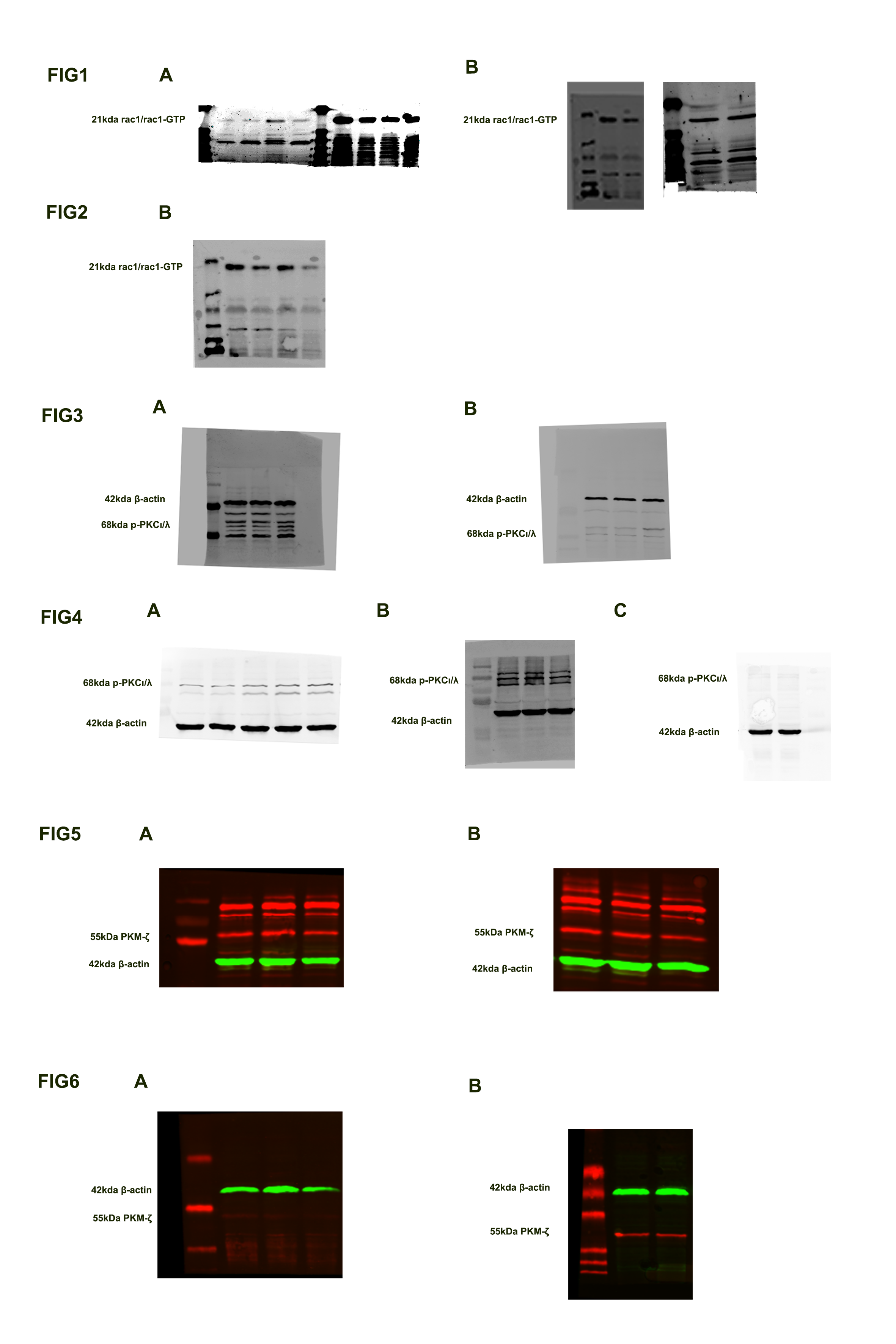

Supplement: Supplementary Figure 2 — The full-length immunoblot. [file Image_2.TIF]
